# Generation and Transport of Dielectric Droplets along Microchannels by Corona Discharge

**DOI:** 10.3390/mi11020181

**Published:** 2020-02-10

**Authors:** Qiang Tang, Shangru Zhou, Ruiheng Hu, Huai Zheng, Junheng Pan, Jau Tang

**Affiliations:** 1 Institute of Technological Sciences, Wuhan University, Wuhan 430072, China; 2 Department of Mechanical and Electronic Engineering, Changsha University, Changsha 410083, China; 3 School of Power and Mechanical Engineering, Wuhan University, Wuhan 430072, China

**Keywords:** corona discharge, microfluidic, microchannel, electroconvection

## Abstract

In this paper, a phenomenon of generation and transport of droplets is proposed, which is based on the dielectric liquid electroconvection induced by corona discharge. We placed the dielectric fluid on a conductive/nonconductive substrate, and then it broke apart to become many small droplets that move along the conductive microchannel. The behaviors of dielectric droplets were experimentally observed on different conductive microchannels in details. Spreading speeds and sizes of dielectric droplets were analyzed at different driving voltages and conductive microchannels. This work highlights a simple approach to produce and manipulate dielectric droplets along microchannels.

## 1. Introduction

Droplet-based microfluidic systems have received significant attention in the past 20 years owing to their low-volume and flexible transmission, which has been found wide applications in miniaturized bio-analytical and chemical fields [1,2,3,4]. The generation and transport of dielectric droplets are critical components of droplet-based microfluidic systems. Droplets have been produced mostly in a passive fashion, such as a T-junction [5] or flow-focusing configuration [6]. Although these technologies show excellent capability in obtaining monodisperse droplets, the major drawback of passive control is the slow response time in the order of seconds or even minutes. Therefore, droplets generation technologies have been developed by using external forces such as magnetic force [7], electric force [8], or acoustic force [9]. Recently, dielectric liquid approach by corona discharge has attracted much attention because of its promising potentials in many different fields [10,11,12,13].

Corona discharge occurs when a high voltage is applied between two electrodes, resulting in partial ionization of the dielectric around the electrode with sharp curvature. Charged carriers (electrons and ions) collide with neutral gas molecules under the influence of an electric field to drive the air flow [14]. The flow generated is generally referred to as electric wind or ion wind. Various patterns of three-dimensional electroconvective flow induced by ion wind in dielectric liquids have been investigated [15,16,17,18,19], especially the regular static cell structures [15,20,21]. At the same time, a number of previous reports refer this to the directional driving of the electric wind exerting upon the dielectric fluid [13,22]. However, the operation and control of microdroplets by corona discharge are rarely mentioned.

Here, we report a phenomenon of corona discharge on a patterned indium tin oxide (ITO) substrate, which forces the dielectric fluid to form small droplets and to transport along conductive microchannels. The small uniform droplets are formed by the electroconvection of the dielectric liquid induced by the corona discharge of needle-plate electrodes. The dielectric droplets can be transported along the microchannel and the size of which can be controlled by the channel width. Various microchannel patterns have been adopted to control the movement of droplets in the presence of corona discharge.

## 2. Materials and Methods

**Figure 1:**
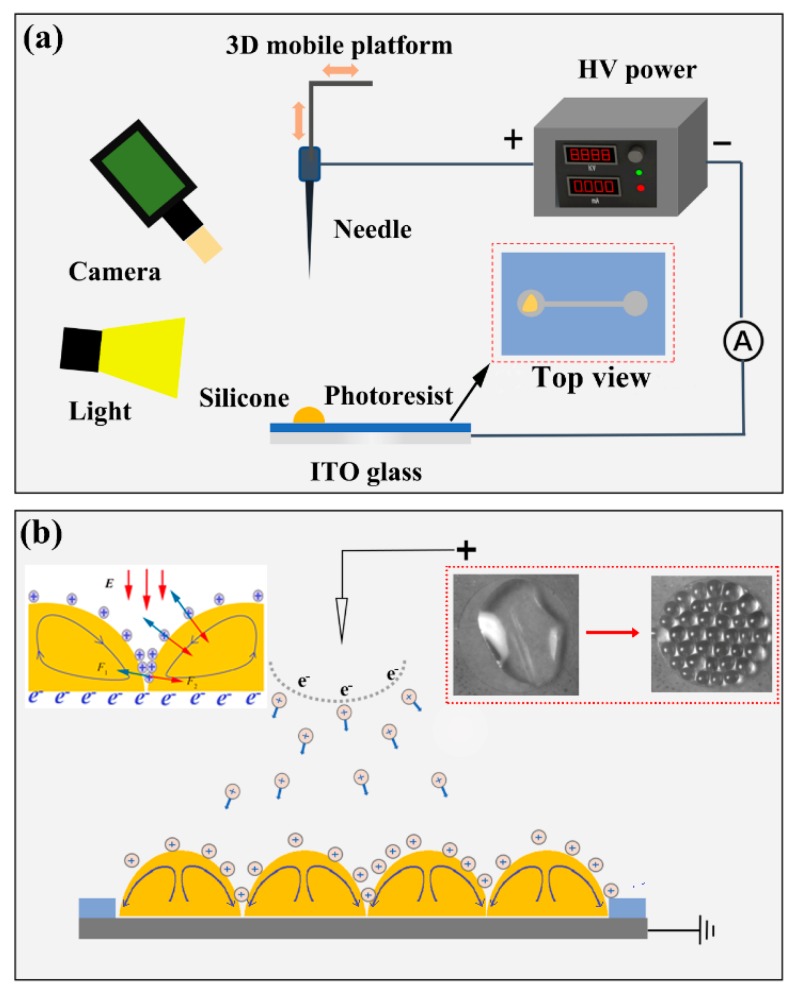
Experimental setup and formation principle of dielectric droplets by ion wind. (**a**) Schematic diagram for the experimental setup. (**b**) Formation principle of dielectric droplets.

The experimental setup for investigating the behavior of dielectric fluid in a microchannel is shown in Figure 1a. The corona discharge is generated from the needle-plate electrodes under a high direct current (DC) voltage. The curvature radius of the tungsten needle tip is about 30 µm, which is connected with the positive electrode of a high DC voltage (DW-P303-5ACCC, Dongwen Corp., Tianjin, China). A micro three-dimensional motion platform is built to control the motion of the needle electrode. The bottom plate with the thicknesses of 0.5 mm is a glass plate covered with transparent ITO (thickness of 185 nm) as the ground electrode. The distance between the needle and the ITO plate is 4.5 cm. The microchannels on ITO were made by photolithography using a positive photoresist (S1813, Dow Rohm&Haas, Midland, TX, USA). The thicknesses of the photoresist films were controlled to be approximately 1.4 μm. Liquid silicone (OE-6650, Dow Corning, Midland, TX, USA) was adopted as the dielectric fluid. Its viscosity, electrical conductivity, and surface tension are about 4.0 Pa·s, 10−8 μS/cm, and 0.021 N/m, respectively. A high speed camera (C13440, Hamamatsu, Shizuoka, Japan) was used to record the dielectric droplets flow in microchannels.

Figure 1b illustrates the motion principle of dielectric fluid droplets along microchannels by the ionic wind. When the voltage difference between the needle and plate electrode exceeds a threshold value such that the electric field strength in the vicinity of the needle becomes sufficiently large to ionize the gas molecules to produce positive or negative ions. The positive ions drift toward the grounded substrate and to deposit charges onto the dielectric droplets. Interactions of the strong electric field and the surface charges deposited on the droplet produces an interfacial electrical pressure. The electric force drives the liquid silicone convection in the vertical direction, and then internal motion takes place and evolves into a self-organized regular pattern of convective cells [23]. During corona discharge, a large number of charges are attached to the liquid surface. Under the action of electric field force, the liquid surface is squeezed. The pressure is vertical to the liquid surface, and the liquid surface has partial depression. Due to the effect of surface tension, the resultant force of tension is perpendicular to the surface. At last, the vertex in the depression almost touches the ITO surface, where the charge is neutralized. The positive charge continuously drives the surface liquid molecules down the surface. After neutralization, it continues to move towards the top of the liquid to induce convection. Finally, the small dielectric droplets were formed. The induced electroconvection with the needle plate configuration presents a clear analogy to the classical Rayleigh-Bénard convection, often observed in a fluid with a temperature gradient [24]. Conductive areas on substrates are transmission paths for ions. Thus, dielectric droplets with ions flow toward microchannels under the action of the electric force.

## 3. Results

**Figure 2:**
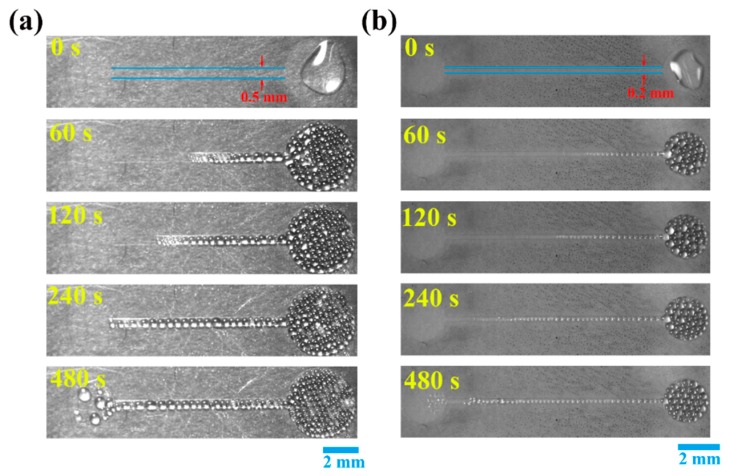
Dielectric droplets flow along microchannels under the action of corona discharge. (**a**) The channel width is 0.5 mm. (**b**) Similarly but with a channel width of 0.2 mm.

Figure 2 shows examples of the photographic sequences of evolution of dielectric droplets driven by corona discharge in two kinds of microchannels at a voltage bias of 8 kV. An irregular shape liquid silicone droplet with a volume of 20 µL is placed on the conductive circular area under the needle, the dielectric droplets formed and flow into the microchannel when the needle tip is electrified. As shown in Figure 2a, the radius of the circular area is 2 mm, and the length of the microchannel is 10 mm. The dielectric droplets flow in the channel orderly and then fill the entire conductive field. In Figure 2b, the radius of the circular area is 1 mm, and the length of the microchannel is 10 mm. The same phenomenon has also occurred. The difference is the dielectric droplets on the channel in Figure 2a is bigger than it in Figure 2b. Such a phenomenon is different from electrowetting, where dielectric droplets were deformed and displaced by changing the wettability of droplets.

**Figure 3:**
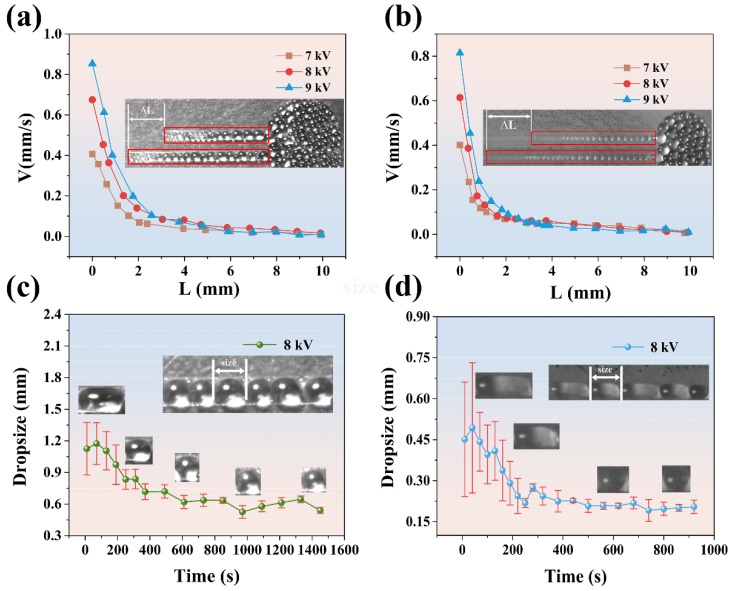
The speed and dielectric droplets size in microchannels. (**a**) The speed of dielectric droplets in channel width 0.5 mm. (**b**) The speed of dielectric droplets in channel width 0.2 mm. (**c**) The size of dielectric droplets in channel width 0.5 mm. (**d**) The size of dielectric droplets in channel width 0.2 mm.

In order to study the flow law of dielectric droplets along microchannels, the spreading speed of silicone was analyzed systematically. Figure 3a,b show the spreading rates at different spreading distances and different driving voltages on two types of microchannels. In the figure, the spreading speed was calculated using the following equation:

(1) $$\begin{array}{c} v=\frac{\Delta L_{L}}{\Delta t_{L}} \end{array}$$

where *v* is the spreading speed and $\Delta L_{L}$ is the variation of the liquid precursor interface location in the continuous two pictures recorded by a high speed camera. $\Delta t_{L}\;$is the time interval between the continuous two pictures, and it is about 1 s. From this figure, it can be seen that the flow velocity of the two kinds of channels decreases rapidly at first and then changes slightly. By comparing the spreading speed at different voltages, it can be found that when the spreading distance is short (<2 cm), the effect of voltage on the diffusion speed is very obvious. The higher voltage leads to higher initial flow velocity. The highest spreading speed on the two kinds of channels is about 8.5 mm/s and 8.1 mm/s at the beginning of the flow and the driving voltage of 9.0 kV. In addition, all droplets in ITO are reversible at any time. When the power is off, the droplets will flatten in place and become liquid. When the power is turned on again, it will return to its original state.

In addition, we also analyzed that the change of averaged dielectric droplets size in the microchannels at a driving voltage of 8.0 kV, as shown in Figure 3c,d. All error bars in this paper are Standard Error. From the figure, it can be seen that the size of dielectric droplets in microchannels becomes smaller and uniform with the increasing spreading time. Finally, the size of the dielectric droplet was kept about the same with the width of the microchannel. It shows that the width of the channel can influence the size of the droplets in the channel at an appropriate voltage.

**Figure 4:**
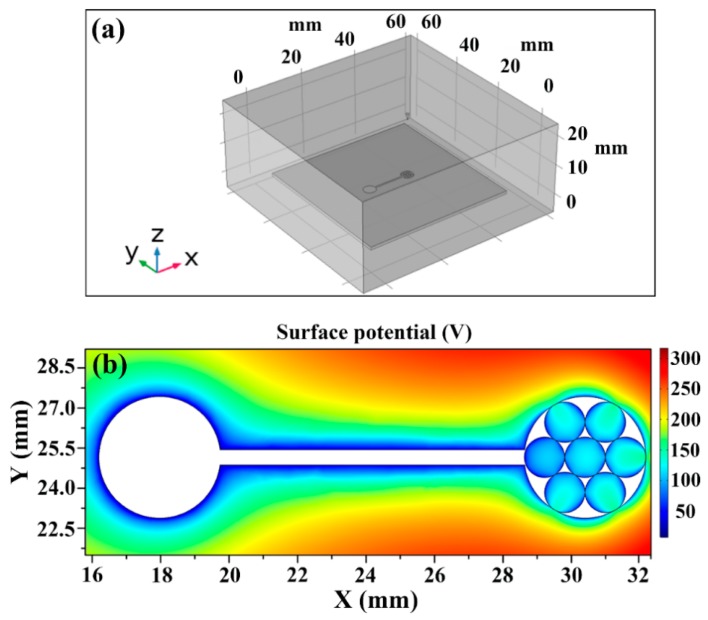
(**a**) Simulation model. (**b**) Electric potential simulation on the ITO film surface.

To evaluate the main factors affecting droplets’ movement and formation in the study, the electric field of droplets on the ITO film surface was simulated using the electrostatic modules of COMSOL Multiphysics (version 5.3a, COMSOL Co., Ltd., Shanghai, China). Experimental simulation model in Figure 4a was made according to the actual experimental parameters, and the voltage applied to the needle tip was 8 kV. In Figure 4b, the simulation result of electric potential on the ITO film surface is shown. At a micrometer scale, viscosity and surface tension should strongly influence the fluid behavior. So in this study, several parameters of fluid flow in microchannels should be considered. The Reynolds number Re, for comparing inertia and viscosity, can be written as

(2) $$\begin{array}{c} R_{e}=\frac{\rho vl}{\eta }\sim 10^{-6} \end{array}$$

where *η* is the viscosity of the silicone (≈4.0 Pa.s), ρ is the density of the silicone (≈0.93 × 10^3^ kg/m^3^), *v* is the velocity of a dielectric droplet (~10^−4^ m/s), and *l* is the width of a channel (~10^−4^ m). The Reynolds number shows that inertia has little effect on droplets motion in microchannels. The capillary number$\;C_{a}$, for comparing viscosity and surface tension, is written as

(3) $$\begin{array}{c} C_{a}=\frac{\eta v}{\gamma }\sim 10^{-2} \end{array}$$

γ is the surface tension between air and dielectric drop (0.021 N/m). And the electric capillary number$\;C_{e}$, which represents the ratio of electric stress ($\epsilon_{L}\epsilon_{0}E^{2}$) and capillary stress (γ/r), for comparing electrostatic forces and surface tension, is written as [25]

(4) $$\begin{array}{c} \;C_{e}=\frac{\epsilon_{L}\epsilon_{0}E^{2}r}{\gamma }\sim 10^{2} \end{array}$$

where $\epsilon_{L}$ is the dielectric constant of liquid silicone, $\epsilon_{0}$ is the permittivity of vacuum, r is the size of the dielectric droplet (~10^−4^ m), E is the electrostatic field around a dielectric droplet (~100 V, as shown in Figure 3b). These considerations suggest that the formation of small droplets is not related to the surface tension of the dielectric liquid, and the electrostatic forces around the droplets play a decisive role in fluid deformation and motion.

**Figure 5:**
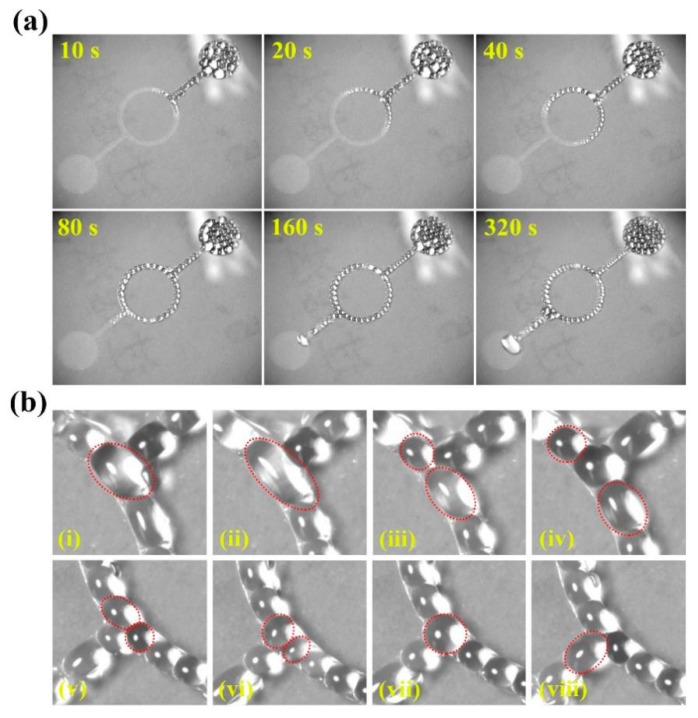
The dielectric droplets flow along curved microchannels. (**a**) Photographic sequences of the evolution of dielectric droplets in curved microchannels (**b**) The split of droplets at the beginning of circular channel (i), (ii), (iii) and (iv), the coalescence of droplets at the end of circular channel (v), (vi), (vii) and (viii).

Besides directional spreading along a straight channel, the corona discharge can drive the silicone spreading along the specific curved channel. Figure 5a shows that the silicone flow along the curved channel of lithography, which driven voltage is 8.0 kV, and the channel width is 0.5 mm. The curved channel is to add a circular channel in the middle of the original channel, with a width of 0.5 cm and an inner diameter of 3 cm. From this figure, it can be seen that dielectric droplets flow from a straight channel to a circular channel and then fill the entire channel pattern. It shows that photolithography channels with different patterns can control the flow direction of dielectric droplets. Furthermore, it can also be found the splitting and coalescence of droplets in the microchannel, as shown in Figure 5b. The photographic sequences of (i), (ii), (iii) and (iv) are the processes of droplet splitting. When the dielectric droplet enters the circular channel, it became elongated and then was split into two droplets moving along the upper and lower channels, respectively. And the photographic sequences of (v), (vi), (vii) and (viii) are that when two droplets of the dielectric fluid touch, they merge into one droplet, driven by Coulomb force. So this provides a way for microfluidics to split and merge droplets under corona discharge.

**Figure 6:**
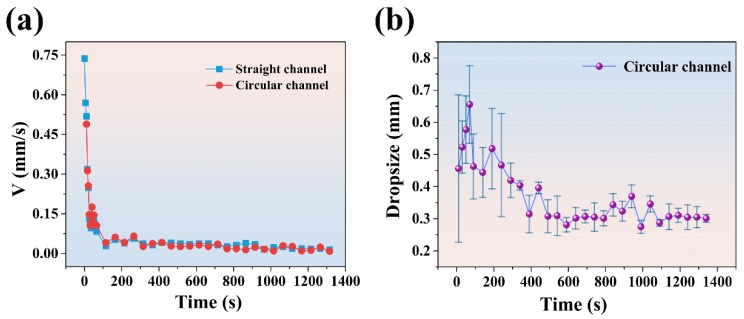
(**a**) The speed of dielectric droplets in the circular channel and straight channel (**b**) The size of dielectric droplets in the circular channel.

By comparing the velocity of droplets along a straight channel with that along a circular channel, as shown in Figure 6a. It can be found that the droplets in a straight channel can maintain the original speed. And the velocity of droplets in different positions of the channel is similar at the same time. Figure 6b shows the change of the average size of droplets in the circular channel, and it can be observed that the sizes of droplets are large and uneven in the early stage, and then become uniform and remain at 0.3 mm. The size of the droplets is different from the size in Figure 2a. That is because the droplet split after flowing into the circular channel, as shown in Figure 5b.

## 4. Discussion and Conclusions

In conclusion, a method of producing uniform dielectric droplets of 0.2 mm to 0.5 mm on microchannels by corona discharge was demonstrated and characterized in details. The size of droplets can be adjusted by the microchannel width, and the moving speed of droplets along the microchannel at the beginning varies with the driving voltage. And one can also split and merge the dielectric droplets through the different patterns of microchannels. This work offers insights into the mechanism of producing and operating small uniform dielectric droplets, which is thought to be a big challenge due to its low surface tension of the dielectric fluid. With a better understanding of the physical mechanism, one could develop microfluidic techniques to more precisely control droplet’s size and movement in potential industrial applications.
